# Adapting the 3D-printed Openflexure microscope enables computational super-resolution imaging

**DOI:** 10.12688/f1000research.21294.1

**Published:** 2019-11-26

**Authors:** Stephen D. Grant, Gemma S. Cairns, Jordan Wistuba, Brian R. Patton

**Affiliations:** 1 Department of Physics and SUPA, University of Strathclyde, Glasgow, UK

**Keywords:** Light Microscopy, Super-resolution, Open Science, 3D Printing

## Abstract

We report on a 3D printed microscope, based on a design by the Openflexure project, that uses low cost components to perform fluorescence imaging. The system is sufficiently sensitive and mechanically stable to allow the use of the Super Resolution Radial Fluctuations algorithm to obtain images with resolution better than the diffraction limit. Due to the low-cost components, the entire system can be built for approximately $1200.

## Introduction

The Openflexure microscope is an open-access, 3D printable microscope which has three dimensional movement of the specimen stage provided by a flexible plastic mechanism ^1^. It uses easily obtainable components and the fine adjustment and stability of the 3D printed microscope allows high quality optical images to be collected. Standard versions of the microscope use medium to low-resolution lenses, enabling observation of cellular features of sub-micron size.

Super-resolution radial fluctuations (SRRF) allows super resolution information to be extracted from a series of fluorescence images taken with a high numerical-aperture (NA) lens (typically NA>0.8). SRRF is a post-processing method that generates a radiality map for each frame and then looks for temporal correlations across frames to create a final image of greater resolution than the original images ^2^. The final resolution achievable by SRRF depends on multiple factors, including the strength of the fluctuations used to generate the correlations, the labelling density and the photo-stability of the fluorophores; however, in the best-case scenario it shows resolution approaching that of localisation based super-resolution methods while utilising a wider range of hardware and fluorophores. Low illumination intensities also avoid photo-bleaching samples ^3^.

Combining SRRF with an adapted version of the Openflexure 3D printable microscope allowed for high quality super resolution imaging on low cost hardware. SRRF analysis was carried out using ImageJ ^4, 5^ plugins meaning all aspects of this work (SRRF algorithms, Microscope schematics, etc.) are freely available and open-access.

## Hardware design

**Figure 1.**
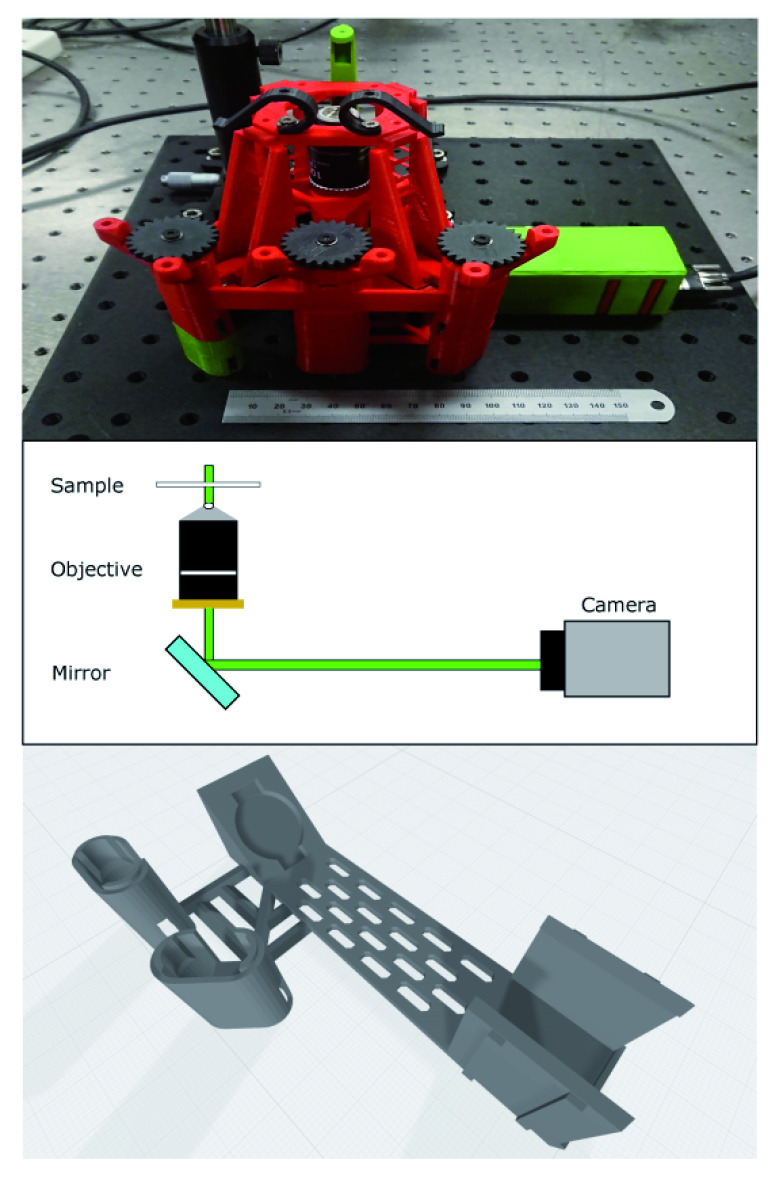
Layout of microscope. Top - image of microscope as constructed. It is mounted on an aluminium breadboard for portability and stability. The ruler is 15cm and shown for scale. The colours of individual parts are arbitrary. Middle - beam path showing key design parameters. Illumination is from a laser mounted above the sample and the camera is mounted at the primary imaging plane of the microscope objective with an emission filter directly attached. The turning mirror allows a more compact design. Bottom: integrated mirror and camera mount to allow imaging with high sensitivity camera. We used the slots in the base of this part to pass mounting screws to the breadboard for additional stability.

We give links to the designs for all key components at the end of this paper (See underlying data ^6^ and hardware design). Measurements were performed using an adapted version of the open source 3D printable microscope (v5.17.2-LS75-M) designed by the Openflexure project. The upper image in Figure 1 shows the microscope as constructed and mounted on a 30×30 cm aluminium breadboard for stability and portability. The colour scheme is arbitrary and does not contain any information about which parts are custom. The custom parts for this project consist of a mirror and camera holder beneath the microscope along with taller legs to allow space for the new optical path shown in the middle of Figure 1. The mirror and camera holder are combined into a single piece to control the distance from the objective to the camera and make the optical alignment more robust. This new piece is shown in the lower image of Figure 1. We also designed a cover for the camera unit to block external light and thereby improve signal to noise. The microscope was printed using polylactic acid material from Ultimaker (Red PLA - Ultimaker part no. 1618 and Green PLA - Ultimaker part no. 1608) on an Ultimaker S5 printer. As our modifications are based on a standard version of the Openflexure stage, stepper motors can be attached to the microscope if required. In this instance all stage movement and focus adjustments were made by hand with the attached adjustment gears. A compact laser module from Thorlabs (CPS532a: 4.5 mW @ 532nm) was used as the light source. This was focused using a Thorlabs plano-convex lens (LA1461-A: f= 250 mm), which gives a minimum spot size of 19 *μ*m at the focus. We purposely defocussed this lens to give a wider illumination spot. The excitation wavelength was chosen to allow us to image the fluorescent Nitrogen-Vacancy (NV) defect centre in samples containing nanodiamond.

A Nikon oil immersion objective (NA=1.25, 100×, Edmund Optics part number #59-938) was used to image the sample directly - this objective is not an infinity corrected lens, and so the camera is placed at the primary image plane. The objective was mounted beneath the microscope stage. Focus adjustments are performed by moving the objective vertically using the adjustment gears as is standard with the Openflexure system.

A Thorlabs silver mirror (PF10-03-P01) was placed beneath the objective in our custom holder and directs the beam onto the camera. The microscope uses a UI-3060CP-M-GL camera from IDS connected to a laptop computer to capture images. The IDS camera sensor (Sony IMX174LLJ-C) has dimensions of 11.345 mm × 7.126 mm with pixel size 5.86 × 5.86 *μ*m. As printed, our mirror and camera mount places the camera sensor 170mm from the mounting flange of the lens. This is not in accordance with the DIN standard of 150mm. However, for the samples used we found that it did not create significant imaging errors but does require calibration of the image field of view at the sensor. By imaging a chrome grid sample with 10 *μ*m grid spacing, we were able to determine that we have a field of view of approximately 95.8×60.2 *μ*m within the sample. This corresponds to a 49.5nm pixel size at the sample; we are sampling above the Nyquist criterion for our optical system. With the known physical pixel dimensions for the camera, we therefore have a magnification of 118x rather than the 100x from the lens specifications.

To reject excitation laser light and allow only the emission from the NV centres to be detected, we used a 650nm longpass filter (Thorlabs FELH0650) mounted directly to the camera’s c-mount hardware.

## Sample preparation

We used two types of sample for this paper and their preparation is as follows:

### Nanodiamond on coverslips

We used samples previously described in 7 and the following preparation protocol is from this reference. The nanodiamonds (ND) used in these experiments were produced by Adamas (Catalogue numbers ND-NV-40nm-Bio-2mg and ND-NV-100nm-COOH-2ml). We prepared slides for imaging from a mix of two monodisperse suspensions (both 0.1%w/v) of 40nm and 100nm diameter ND. The 40nm ND each contain approximately 10 NV while there are closer to 400 NV per 100nm ND as per the manufacturers calibration information. To prepare a suspension suitable for deposition on a coverslip we first sonicated each source of NDs at room temperature for approximately 30 minutes (we have not found this step to be time critical), using a Grant Ultrasonic Bath XUBA3, to break up larger aggregates before adding 10 *μl* of each ND suspension to 100 *μl* of distilled water. The resulting suspension was then deposited onto a #1.5 microscope glass cover slip and allowed to dry to ensure some ND adhered to the coverslip before being mounted on to a microscope slide using a small amount of distilled water as a mountant medium and finally sealing the sample with nail polish.

### Monocyte-derived macrophages

Whole blood samples were obtained, after written informed consent, from healthy donors on the Centre for Inflammation Research Blood Resource (approved by AMREC, reference number 15-HV-013). Peripheral blood mononuclear cells (PBMCs) were first isolated from these samples before being plated onto #1.5 square glass coverslips (22×22mm) in 6 well plates at a concentration of 9 *×* 10 ^6^ cells/coverslip. The PBMCs were then cultured in media (RPMI (Sigma Aldrich catalogue R8758) containing 10% low lipopolysaccharide (LPS) fetal calf serum (FBS Good Forte, Pan Biotech, P40-47500) in an incubator at 37 *°*C with 5% carbon dioxide ( *CO*_2_). They were cultured for 14 days with media changed twice per week to allow for the differentiation of PBMCs into monocyte-derived macrophages (MDMs).

90nm nanodiamonds (Sigma-Aldrich 798150) were sonicated for 1 hour to break up aggregates and then added to media at a dilution of 1:100. After 14 days of cell culture, MDMs were incubated with 1 ml of the nanodiamond/media solution at 37 *°*C with 5% *CO*_2_ for 2 days. After 2 days, media was removed from the MDMs before washing three times with pre-warmed Hank’s balanced salt solution (with calcium and magnesium) to remove any remaining media. MDMs were then fixed using 2% paraformaldehyde (PFA) for 20 minutes at room temperature. Finally, the MDMs were washed three times with phosphate buffered saline (PBS) and once with deionised water ( *dH*_2_ *O*) before mounting the coverslips onto microscopy slides with ProLong Diamond Antifade Mountant(Thermofisher catalog number P36970).

## Data acquisition and analysis

Data was collected using the supplied IDS software (IDS Software Suite 4.92) with gamma, auto exposure, and auto gain switched "off". Exposure time and frame rate were adjusted to produce an image with signal in the middle of the 8-bit greyscale range. Stacks of 200 images were captured as 200 frame videos. These stacks were then processed using nanoJ-Core V2.1 RC1 ^8^ to determine the drift correction and nanoJ-SRRF v1.14Stable1 ^3^ to preform super resolution radial fluctuations analysis. For the analysis of all the data presented here we used the following processing parameters within the Fiji plugins.

- Drift Correction: Averaged drift over 5 frames and corrected position to first frame
- SRRF: Ring radius 0.5, Radiality Magnification 6, Axes in Ring 7. Other parameters were default for plugin.

Images are presented using the Cube Helix colormap ^9^ and were generated for publication using OriginLab Origin.

All raw (unprocessed) image data is available along with the microscope design files (Underlying data ^6^).

## Results

**Figure 2.**
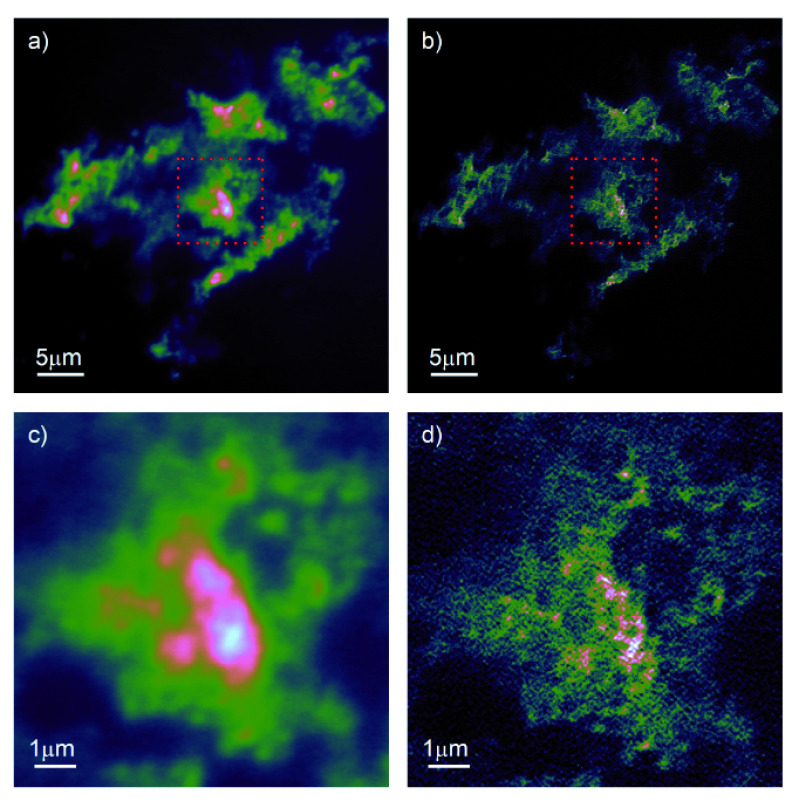
Fluorescence imaging of 40nm and 100nm nanodiamond on coverslip. **a**) Drift corrected and summed stack of 200 individual frames. **b**) Superresolution radial fluctuations (SRRF)-processed image of same stack of images. **c**), **d**) zoom on equivalent region marked by red dashed box in **a**), **b**) showing increased resolution enabled by SRRF.

Figure 2 shows the results of imaging ND directly mounted on a coverslip. In the standard fluorescence image, Figure 2a), we see good contrast, particularly after correcting for sample drift and summing the frames. A significant increase in resolution is visible after processing the stack using the SRRF algorithm, Figure 2b). To better demonstrate the effect of the image processing, we have expanded the region in the red dashed box and present it in Figure 2c) and d). We can now clearly see individual NDs, particularly at the edges of the region highlighted. In the central region, it is likely that some of the structure shown is due to single ND; however, we cannot currently unambiguously rule out the presence of artefacts from the image processing algorithm.

In order to better understand the resolution enhancement, we processed the images using the Fourier Ring Correlation technique (FRC) ^10^. To estimate resolution, FRC processes pairs of images that differ only in noise: at low spatial frequencies which are dominated by the (identical) structure of the sample there is a high degree, *FRC* ≈ 1, of correlation, which decreases with increasing spatial frequency until reaching a point at which the images are dominated entirely by uncorrelated noise. The spatial frequency at which this occurs is considered to be equivalent to the effective resolution of the image. There are two important caveats here:

1. The image may possess no significant features at the resolution of the system generating the image. In this case, the FRC will return an apparent resolution worse than the actual system resolution.
2. The FRC does not actually measure resolution directly, but maps spatial frequency correlations to resolution.

To consider why the second point is of relevance for our paper here, consider performing the FRC of an image, consisting entirely of random pixel values (pixel noise), with itself. In this case, the FRC would correctly return a perfect correlation at all spatial scales, and any implications on the resolution of the system would be meaningless. This incorrect resolution estimation can also arise as the signal to noise ratio (SNR) can itself influence the apparent resolution returned by the FRC algorithm. In most fluorescent microscopies, this is not a significant issue, as the fluorophores degrade and so put a limit on the maximum SNR obtainable from the sample. However, the fact that the ND emission does not degrade means that we can achieve arbitrarily high SNR by either increasing the exposure time of our sensor, or summing more sub-frames. When calculating the FRC for Figure 2a) we ran into this problem with the SNR; if you calculate the FRC by performing the correlation on the summed, drift-corrected odd versus even frames the FRC implies super-resolution imaging in wide field mode! By reducing the number of sub-frames used for the FRC, we obtained a resolution of 238 nm, which is in line with the expected diffraction limited resolution for a wide-field microscope using a 1.25NA objective lens, albeit implying a higher resolution than the 280nm expected for the ND emission peak at 700 nm. An alternative approach to estimating the resolution is to measure the full width half maximum (FWHM) of individual emitters within the image. Due to the background, it is difficult to unambiguously determine the resolution in this sample. However, measuring the resolution in this manner consistently returned values for the FWHM in the range of 300–400 nm, i.e. close to diffraction limited performance.

The FRC of the SRRF processed images generated correlation curves that followed the expected behaviour for reliable resolution estimation. In this case, we recover an FRC resolution of 115 nm. Again, this is slightly higher than the resolution returned by FWHM measurements, which tended to the range 120–140 nm. Nevertheless, it supports our assertion that the modifications to the microscope allow computational super-resolution imaging using the SRRF technique.

**Figure 3.**
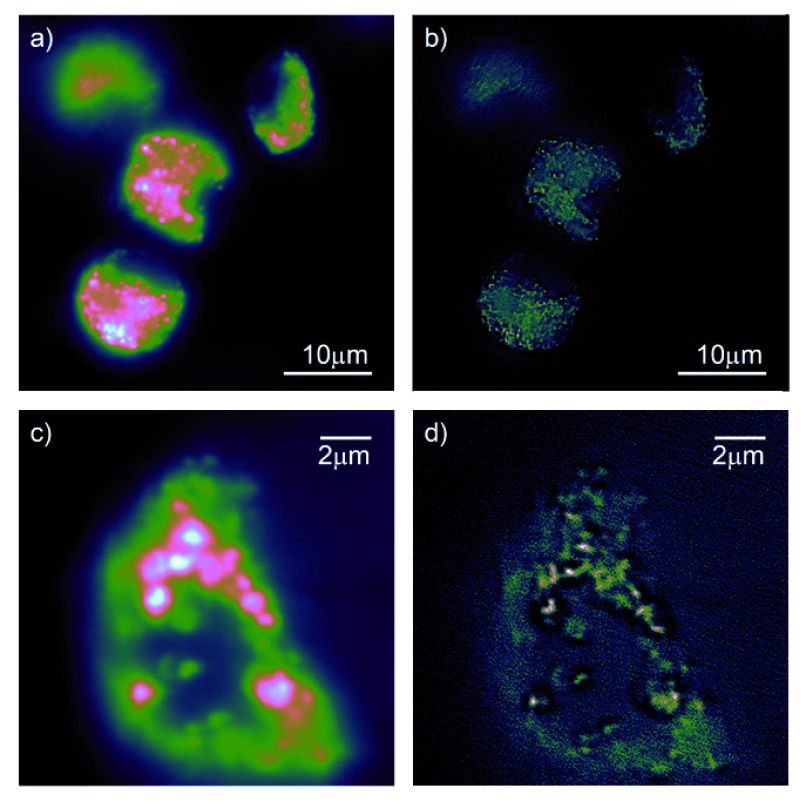
Fluorescence imaging of 90 nm nanodiamond in monocyte-derived macrophages. The nanodiamonds were suspended in the growth medium before uptake by the macrophages. **a**), **b**) large field of view encompassing multiple macrophages in both wide-field fluorescence and super-resolution radial fluctuations (SRRF) imaging modes. **c**), **d**) detail view of single macrophage in both widefield and SRRF modes.

Imaging bright, stable fluorophores that are confined to the surface of a cover slip can be considered to be the optimal sample for the characterisation of a microscope system and so somewhat unrealistic. We therefore also used ND in MDM as described above, and the resulting widefield and SRRF images are shown in Figure 3 a)-d). Again we see that the microscope performs well, generating high quality fluorescence images that process well with the SRRF algorithm. The slightly lower SNR in these images returned more reliable FRC estimates, and we see FRC resolution of 322nm for the widefield images and 114nm for the SRRF images.

## Conclusions

SRRF imaging is possible using low cost, open-access components and software on a 3D printed microscope, achieving resolutions of 115 nm, compared to the unprocessed (standard) resolution of 322 nm.

When compared to traditional optical systems capable of super resolution, SRRF presents a much simpler method while the instrumentation shown here allows for its implementation at much lower costs.

## Data availability

### Underlying data

A zip file containing the 3D printable .STL files and the raw (unprocessed) data used in the images in this paper is available from the University of Strathclyde institutional repository

University of Strathclyde Knowledgebase: Data for “Adapting the 3D-printed Openflexure microscope enables computational super-resolution imaging” https://doi.org/10.15129/df032aa8-2b85-4ec8-adf8-ad435806b81b ^6^

This project contains the following underlying data:

- Folder: Figure-Layout (Contains files used for Figure 1)
  – BetterMicroscopeImage.JPG (A JPEG photograph of the microscope
  – Cam_MirrorSupportWithLegs_V3.1.png The rendering of the custom mirror and camera unit in PNG format)
  – Figure-LayoutV2.pdf (The file used to generate Figure 1, in PDF format)
  – Figure-LayoutV2.svg (The file used to generate Figure 1, in SVG format)

- Folder: Figure NV (Contains files used for Figure 2)
  – FigureNDonCoverslip4.pdf (The file used to generate Figure 2 in PDF format)
  – FigureNDonCoverslip4.png (The file used to generate Figure 2 in PNG format)
  – NV200Frames4.avi (The movie (in AVI format) containing the raw data for Figure 2)

- Folder: Figure-Macrophages (Contains files used for Figure 3)
  – Figure-Macrophages.png (The file used to generate Figure 3, in PNG format)
  – MacrophageFrames3.avi (The movie (in AVI format) containing the raw data for Figure 3)

- Folder: Microscope STL Files (This folder contains multiple .stl files that can be directly printed on a 3D printer that can print files in this widely used file format. All parts should be printed and assembled using the instructions on the https://openflexure.org/ website, along with the additional details given in this paper)

All figures and raw data are available under the terms of the Creative Commons Attribution 4.0 International license (CC-BY 4.0).

The .stl files for the 3D printer, including the new components described in this paper, are available under the terms of the CERN Open Hardware licence (CERN OHL).

## Hardware design

The microscope was constructed using an adapted version of the open source 3D printable microscope (v5.17.2-LS75-M) designed by the Openflexure project, https://openflexure.org/, with modifications as described above.

## References

[1] Sharkey JP Foo DC Kabla A: A one-piece 3D printed flexure translation stage for open-source microscopy. *Rev Sci Instrum.* 2016;87(2):025104. 10.1063/1.4941068 26931888

[2] Gustafsson N Culley S Ashdown G: Fast live-cell conventional fluorophore nanoscopy with ImageJ through super-resolution radial fluctuations. *Nat Commun.* 2016;7(1):12471. 10.1038/ncomms12471 27514992 PMC4990649

[3] Culley S Tosheva KL Matos Pereira P: SRRF: Universal live-cell super-resolution microscopy. *Int J Biochem Cell Biol.* 2018;101:74–79. 10.1016/j.biocel.2018.05.014 29852248 PMC6025290

[4] Rueden CT Schindelin J Hiner MC: ImageJ2: ImageJ for the next generation of scientific image data. *BMC Bioinformatics.* 2017;18(1):529. 10.1186/s12859-017-1934-z 29187165 PMC5708080

[5] Schindelin J Arganda-Carreras I Frise E: Fiji: an open-source platform for biological-image analysis. *Nat Methods.* 2012;9(7):676–682. 10.1038/nmeth.2019 22743772 PMC3855844

[6] Patton B: Data for: "Adapting the 3D-printed Openflexure microscope enables computational super-resolution imaging". 2019 10.15129/df032aa8-2b85-4ec8-adf8-ad435806b81b PMC7255852 32518624

[7] Johnstone GE Cairns GS Patton BR: Nanodiamonds enable adaptive-optics enhanced, super-resolution, two-photon excitation microscopy. *R Soc Open Sci.* 2019;6(7):190589. 10.1098/rsos.190589 31417755 PMC6689623

[8] Laine RF Tosheva KL Gustafsson N: NanoJ: A high-performance open-source super-resolution microscopy toolbox. *J Phys D Appl Phys.* 2019;52(16):163001 10.1088/1361-6463/ab0261 PMC7655149 33191949

[9] Green DA: A colour scheme for the display of astronomical intensity images. *Bull Astr Soc India.* 2011;39:289–295. Reference Source

[10] Nieuwenhuizen RP Lidke KA Bates M: Measuring image resolution in optical nanoscopy. *Nat Methods.* 2013;10(6):557–562. 10.1038/nmeth.2448 23624665 PMC4149789

