# Supplementary material for: Adapting the 3D-printed Openflexure microscope enables computational super-resolution imaging
Source: F1000Res. 2019 Nov 26;8:2003. [Version 1] doi: 10.12688/f1000research.21294.1 (PMC7255852; doi:10.12688/f1000research.21294.1)
Supplement: Peer review history

# Peer review: Reviewer response for version 1

**DOI:** 10.5256/f1000research.23450.r63807

**Published:** 2020-05-26

**Authors:** Romain F. Laine

**Affiliations:** 1 University College London, London, UK; 2 Francis Crick Institute, London, UK

Grant *et al.* provides a clever open-source microscopy platform capable of SRRF super-resolution microscopy. Open-source and cheap microscopy developments are currently a hot topic in the field and a number of contributions are available from various labs across the world. 

 My expertise is in the development of Super-resolution microscopy techniques, including hardware and software-based solutions. This includes the SRRF super-resolution technique and I currently work in the lab (Henriques lab) which pioneered the method.

 Typical open-source microscopes designed for high resolution can suffer from a number of drawbacks:

1. Mechanical stability can be poor and compromise the image acquisition by adding motion blur and sample drift
2. Use of typical high NA microscope objectives can be expensive and require alignment of full optical train (objective, tube lens, camera)
3. Sample movement and focus control can be either bulky and/or expensive 

The implementation of Grant *et al.* nicely combines a number of open-source components (3D printed elements, simple opto-mechanics and cheap CMOS camera) that makes it uniquely simple and powerful and provide interesting trade-offs for these common drawbacks:

- Use of the cheap and flexible OpenFlexure system for sample movement control and objective focus control
- Simple, single 3D-printed block assembly for the detection path of the fluorescence microscope
- Use of finite conjugate objective, doing away with tube lens altogether and simplifying significantly the assembly

The super-resolution capability is enabled by SRRF, an approach based on picking up on radial symmetry of single emitter fluorescence pattern and correlated temporal fluctuations of these emitters. I do not know about the photophysics of NV and their blinking properties but a preprint from Narayanasamy *et al.* ^1^ shows their compatibility with super-resolution microscopy. Furthermore, the resolution improvement quoted and the images shown here are convincing. 

 Using SRRF as a super-resolution microscopy technique is a great way to address the aspect of drift since SRRF requires only relatively short acquisition times compared to other super-resolution microscopy techniques such as SMLM and also allows the correction of sample drift at post-acquisition level. Although SRRF does not provide as high a resolution as single-molecule localization microscopy (SMLM), the short acquisition times are more compatible with the plastic stage of OpenFlexure, which may introduce more sample drift than typical metal body assemblies.

 I agree however with Reviewer 1 that some measurements of stability and amplitude of drift on the order of a typical acquisition would be helpful. This could be easily done on single emitters such as commonly used TetraSpeck beads or the single emitter NV that the authors describe in the paper. 

 The use of a finite objective (which is surprisingly cheap: £203 as of May 2020) with a high NA and a decent plan field of view for the price provides a great compromise between price and performance. This work constitutes the first use of finite conjugate objective to super-resolution microscopy to my knowledge.

 I am especially impressed by the quote of a FWHM of 300-400 nm on single emitters, especially since the objective is not quite working at the nominal conjugate. Is there any reason why the system was not designed to be at the nominal conjugate distance? It would be interesting to get some insight into some of the design choices made here for whoever would like to replicate the system (which I may very well do myself at some point in the future). 

 The discussion about FRC is absolutely valid and is a nice addition to the overall idea of the paper.

 A few additional comments however:

- It is true (as reviewer #1 highlighted) that the illumination system is described only superficially, yet it is unavoidable to build an efficient illumination system for a good quality fluorescence microscopy. Also, the downside of using an illumination configuration coming from the top of the objective and therefore contributing to a larger amount of background light onto the detector should be discussed/mentioned
- Since one of the novelty is the use of finite conjugate objective, it would be interesting to characterise it a little further in terms of PSF size and shape (as preliminary done), flatness of the field, sensitivity (I suspect that transmission is poorer than high end objectives) and multi-colour capability (although the latter might be out of scope)

 
**Is the rationale for developing the new method (or application) clearly explained?**

 Yes, it is a nice set of trade-offs introduced here, truly reducing the cost of high resolution microscope to the bare minimum, taking advantage of 3D printing especially, which I have not seen in other open-source microscopy implementations out there.

**Is the description of the method technically sound?**

 Yes, the authors present a couple of experiments showing proof-of-concept and some performance. 

**Are sufficient details provided to allow replication of the method development and its use by others?**

 Yes, generally. STL files and other technical details are all there. Maybe a bit more info about how the illumination was set up would be nice for completeness.

**If any results are presented, are all the source data underlying the results available to ensure full reproducibility?**

 Yes.

**Are the conclusions about the method and its performance adequately supported by the findings presented in the article?**

 Yes, apart from the couple of suggestions of characterisation of mechanical stability and microscope objective performance which would really nail down a full characterisation of the system presented here.

Is the rationale for developing the new method (or application) clearly explained?

Yes

Is the description of the method technically sound?

Yes

Are the conclusions about the method and its performance adequately supported by the findings presented in the article?

Yes

If any results are presented, are all the source data underlying the results available to ensure full reproducibility?

Yes

Are sufficient details provided to allow replication of the method development and its use by others?

Yes

Reviewer Expertise:

My expertise is in the development of Super-resolution microscopy techniques, including hardware and software-based solutions. This includes the SRRF super-resolution technique and I currently work in the lab (Henriques lab) which pioneered the method.

I confirm that I have read this submission and believe that I have an appropriate level of expertise to confirm that it is of an acceptable scientific standard.

## References

[1] : Self-activated photoblinking of nitrogen vacancy centers in nanodiamonds (sandSTORM): A method for rapid single molecule localization microscopy with unlimited observation time. *bioRxiv*.2020; 10.1101/2020.05.20.106716 10.1101/2020.05.20.106716

# Peer review: Reviewer response for version 1

**DOI:** 10.5256/f1000research.23450.r58499

**Published:** 2020-01-14

**Authors:** Richard Bowman

**Affiliations:** 1 Centre for Photonics and Photonic Materials, Department of Physics, University of Bath, Bath, UK

This article describes an open, low-cost, super-resolution imaging system, using nanodiamond probes and the SRRF algorithm to localise individual emitters. It uses the OpenFlexure Microscope as the critical optomechanical mount, responsible for holding the microscope objective and the sample - consequently, most of the mounting and micropositioning hardware in the system can be 3D printed from open, documented designs; the major expenses are the camera, microscope objective, and laser - totalling around $1200.

 I have led the OpenFlexure Microscope project for some years now, and am very excited to see it used for super-resolution imaging. It is particularly pleasing that this imaging is also enabled by open-source software in the form of ImageJ, nanoJ-Core, and nanoJ-SRRF. The authors have done a great job of producing a clear, concise manuscript that describes the system and their characterisation data, that proves the principle of accessible, DIY super-resolution microscopy. I am very happy to endorse the manuscript as scientifically sound.

 I should be clear about what I don't know; I can't claim to be an expert on super-resolution techniques, and so I'm not able to state authoritatively whether I would trust the FRC figures. The authors are commendably clear about the fact that FRC can be confused by excessively high signal-to-noise ratios. While they do state the FRC figures, they also provide the more direct FWHM measure of the point spread function. FWHM gives only slightly worse resolution figures than FRC; the system is still clearly beating the diffraction limit by some margin.

 Overall, I am delighted to see this manuscript, which really advances what is possible using an inexpensive, home-built microscope. I very much hope that others are able to build on this development, and use some of the techniques shown to make novel microscopy instruments accessible, as well as reproducible.

 I have detailed below a few minor improvements to the manuscript that might help someone reproducing the system. I don't believe any of these affect scientific soundness, but they would nonetheless be helpful.

 First paragraph of "hardware design": "insamples" is missing a space in the PDF version. There's also a "fromthe" later on, though both are OK in the web view.

 Figure 1: it would be nice to add the laser diode and emission filter to the middle panel. I understand the laser enters the sample from the top, and it is stated in the text that the emission filter is mounted to the camera's C-mount hardware - but it would be clearer if they appeared in the diagram. Also, I don't think it's stated anywhere how the laser diode is mounted - is that the half-inch post I see in the background of the photo? While it's hardly a scientifically critical detail, it's helpful for anyone reproducing the system.

 Sample Preparation, final paragraph: were the 90nm nanodiamonds functionalised in any way to label specific features of the MDMs? I don't think that's specified if so. If not, do you know where they tend to localise within the cell? It doesn't affect the imaging performance either way, but I'm curious.

 Data acquisition and analysis: it would be nice to know the total elapsed time of the 200-frame video, and ideally the exposure time if that's available. A comment on how far the stage drifted laterally over that time might also be helpful (particularly because there will, presumably, be axial drift that is not corrected for). Do the authors believe drift was the limiting factor in the resolution that was achieved?

 Reference [1] is the most up-to-date paper on the OpenFlexure Microscope currently published, and the authors are absolutely correct in referencing openflexure.org as the most up to date information on the project. However, we have described the microscope recently in a pre-print and I have taken the liberty of including the DOI below ^1^. This version of the microscope is much closer to the one used in the present article than the earlier version described by Sharkey *et al.* ^2^.

Is the rationale for developing the new method (or application) clearly explained?

Yes

Is the description of the method technically sound?

Yes

Are the conclusions about the method and its performance adequately supported by the findings presented in the article?

Yes

If any results are presented, are all the source data underlying the results available to ensure full reproducibility?

Yes

Are sufficient details provided to allow replication of the method development and its use by others?

Yes

Reviewer Expertise:

My expertise is in optical instrumentation, particularly microscope design and optomechanics.  I am not an expert in superresolution, as stated in my report, but am comfortable assessing the instrumentation aspects of the work.

I confirm that I have read this submission and believe that I have an appropriate level of expertise to confirm that it is of an acceptable scientific standard.

## References

[1] : Robotic microscopy for everyone: the OpenFlexure Microscope. *bioRxiv*.2019; 10.1101/861856 10.1101/861856 PMC724983232499936

[2] : A one-piece 3D printed flexure translation stage for open-source microscopy. *Rev Sci Instrum*.2016;87(2) : 10.1063/1.4941068 025104 10.1063/1.4941068 26931888
